# Motor Assisted Commutator to Harness Electronics in Tethered Experiments

**DOI:** 10.1523/ENEURO.0583-24.2025

**Published:** 2025-05-02

**Authors:** Jose Rodriguez-Romaguera, Jovan Tormes-Vaquerano, Ellora M. McTaggart, Maria M. Ortiz-Juza, Noah W. Miller, Kameron Thomas, Antonio Florido, Nicolas C. Pégard

**Affiliations:** ^1^Departments of Psychiatry, University of North Carolina at Chapel Hill, Chapel Hill, North Carolina 27514; ^2^Cell Biology and Physiology, University of North Carolina at Chapel Hill, Chapel Hill, North Carolina 27514; ^3^Neuroscience Center, University of North Carolina at Chapel Hill, Chapel Hill, North Carolina 27514; ^4^Carolina Institute for Development Disorders, University of North Carolina at Chapel Hill, Chapel Hill, North Carolina 27514; ^5^Department of Applied Physical Sciences, University of North Carolina at Chapel Hill, Chapel Hill, North Carolina 27514; ^6^Carolina Stress Initiative, University of North Carolina at Chapel Hill, Chapel Hill, North Carolina 27514; ^7^Neuroscience Curriculum, University of North Carolina at Chapel Hill, Chapel Hill, North Carolina 27514; ^8^Department of Biomedical Engineering, University of North Carolina at Chapel Hill, Chapel Hill, North Carolina 27514

**Keywords:** freely behaving, in vivo, motorized commutator, open source

## Abstract

Research that combines advanced technological devices with complex behavioral tasks has enabled investigations into the neural mechanisms underlying brain and behavioral states. Freely moving rodent experiments often require a tether—a wired connection between an implanted device and an external power supply or data acquisition system. Traditionally, these experiments have used passive commutators to manage tethers, but such setups are often inadequate for reducing twisting and mechanical strain during behavioral tasks. Existing motorized commutators have extended the range of motion for these experiments but generally rely on stepper motors that produce auditory noise, potentially interfering with behavior. To address these limitations, we developed the Motor Assisted Commutator to Harness Electronics in Tethered Experiments (MACHETE), a motor-assisted commutator featuring a low-noise brushless motor. MACHETE dynamically adjusts tethers based on mouse movement, reducing torque and mechanical strain, and minimizing the animal's physical exertion during behavioral assays. Its onboard microcontroller provides customizable controls and seamless integration with custom electronic devices. The design includes a central through-hole to accommodate wires or fibers from external devices such as head-mounted miniature microscopes, electrophysiology probes, and optogenetics systems. We validated MACHETE across standard behavioral assays, including the open field test, the splash test, and the three-chamber social test. Our results showed no significant changes in mobility or behavior compared with untethered controls. By combining precise motor control, low auditory noise equipment, and accessibility, MACHETE can be used to support research that aims to adapt tools for use in freely moving behavior experiments.

## Significance Statement

Experiments with freely behaving rodents in neuroscience often rely on tethered systems to connect animals to data acquisition devices. Existing systems, using passive or stepper motor-driven commutators, are prone to cable twisting, mechanical strain, or auditory interference, potentially affecting experimental outcomes. We introduce MACHETE, a motor-assisted commutator with a low auditory noise brushless motor that enhances tether management, reduces torque, and minimizes behavioral interference. Compatible with widely used electro-optical devices, MACHETE offers a solution for studying brain–behavior relationships in freely moving behavioral tasks. Its adaptability also supports the integration of custom tools for neuroscience research while preserving behavioral integrity in tethered experiments.

## Introduction

To understand how environmental cues are encoded and processed to guide behavior, it is essential to record neural activity in freely behaving animals. Advances in neural recording and modulation technologies—including Neuropixels probes, Miniscope for in vivo calcium imaging, and optical modulation via fiber optic cables—have enabled continuous monitoring and precise manipulation of neural activity in animal models during naturalistic behaviors (Helmchen et al., 2001; Ghosh et al., 2011; Jun et al., 2017; Ozbay et al., 2018; Juavinett et al., 2019; Klioutchnikov et al., 2020; Patel et al., 2020; Voigts et al., 2020; Steinmetz et al., 2021; Zong et al., 2021; Barbera et al., 2024). These innovations have provided valuable insights into the neural circuits of behavior. Building on this progress, considerable efforts have focused on the development of custom devices to adapt techniques traditionally used in head-fixed animals for use in freely moving behavior paradigms (Nodal et al., 2010; Payne and Raymond, 2017; Michaiel et al., 2020). However, widespread adoption of many of these devices in small animals such as mice often remains constrained by challenges related to the weight of devices and the management of wired tethers required for power and data acquisition (Ozbay et al., 2018; Zong et al., 2022; Das et al., 2023).

Wired tether connections are prone to entanglement during freely moving behavior experiments, especially in tasks involving repetitive movement patterns. To mitigate this, many experiments rely on manual intervention by researchers to untangle cables and ensure animal safety or prevent equipment damage (Helmchen et al., 2001; Zong et al., 2022). However, these interruptions can limit naturalistic behavior and disrupt experimentations by introducing behavioral artifacts (Kapanaiah and Kätzel, 2023; Newman et al., 2024; Oladepo et al., 2024). Alternatively, passive, non-motorized commutators, which use flexible, lightweight cables managed by counterweight-pulley systems, are employed (van Daal et al., 2021). While effective for low-activity tasks, these systems are unsuitable for high-activity behavioral assays, as small animals often cannot generate sufficient torque to rotate the commutator effectively (Fee and Leonardo, 2001; Kapanaiah and Kätzel, 2023).

Existing active, motor-assisted commutators address some of these issues and have expanded the range of motion in freely moving experiments (Barbera et al., 2020; De La Crompe et al., 2023; Kapanaiah and Kätzel, 2023). However, many of these devices rely on stepper motors, which generate auditory noise that can impact behavioral results. To overcome this limitation, we developed the Motor Assisted Commutator to Harness Electronics in Tethered Experiments (MACHETE), an open-source 3D-printed solution for freely moving animal behavior experiments. MACHETE employs a brushless motor to minimize noise and reduce torque, enabling smoother tether management during behavioral assessment. An onboard microcontroller permits customizable controls and seamless integration with commercial and custom-built devices. Multiple data ports and a 20-mm-diameter central through-hole design accommodates integration with additional commonly used neuroscience tools.

Here, we provide detailed instructions for assembly and present data showing that mice tethered to MACHETE exhibit no significant behavioral differences compared with untethered controls, with these findings replicated across multiple behavioral assays.

## Materials and Methods

### Motor-assisted commutator

#### Overview

**Figure 1.**
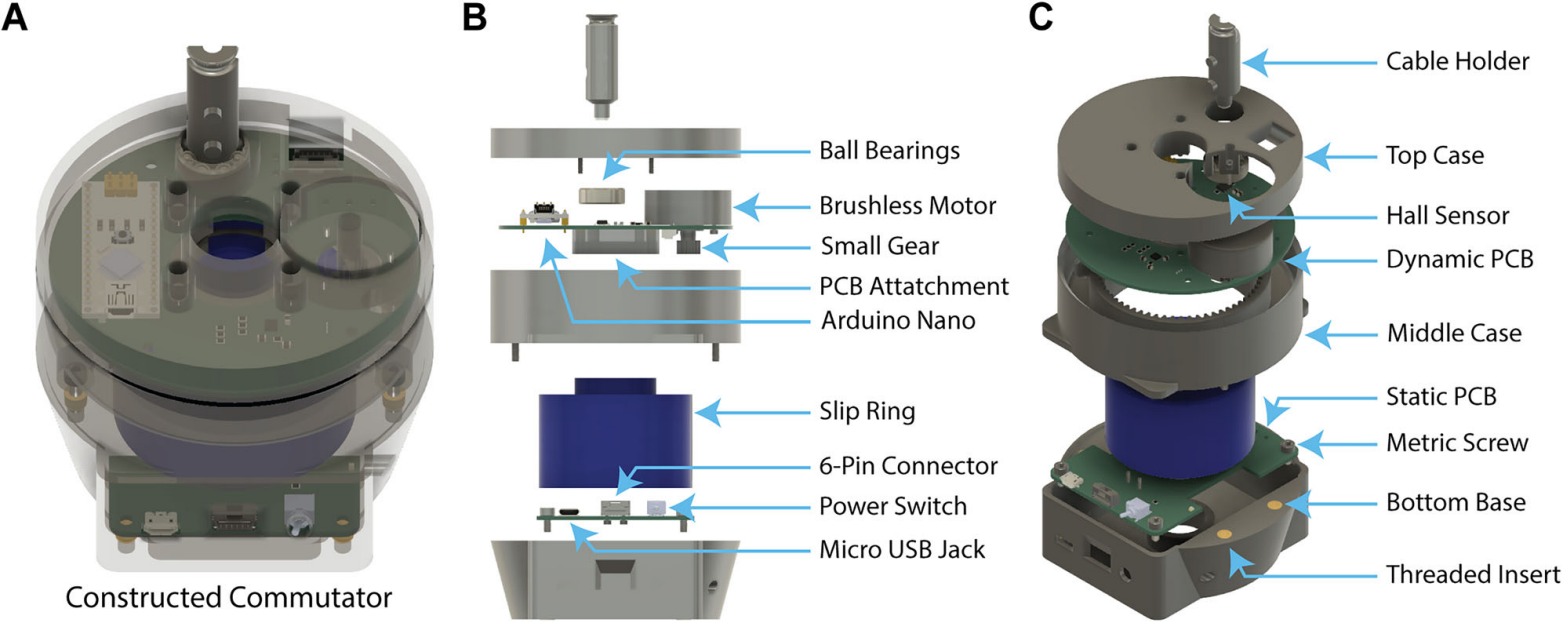
Schematic of MACHETE. ***A***, 3D model of the fully assembled device, displayed with a transparent case to illustrate the layout of internal electromechanical components. ***B***, Exploded front-facing view of the motor-assisted commutator, labeling major internal components and their positions within the assembly. ***C***, Isometric view of the commutator, highlighting additional internal and external components not visible in panel ***B***.

The general architecture of MACHETE is illustrated in Figure 1*A*. MACHETE consists of custom 3D-printed parts, commercially available electromechanical components, and two custom-made printed circuit boards (PCBs; a detailed list of components is provided in Tables 1 and 2). These PCBs are built around a slip ring, which facilitates continuous data and power transmission while actively rotating to relieve torsion in tethered wires.

**Table 1.** List of components of the printed circuit boards for the motor-assisted commutator

Purpose	Item	Supplier/product number	Quantity	Price (US)
Static PCB	Static PCB Board	Fabricated PCB (File: Part6_Static_PCB.zip)	1	0.20
	Micro USB 2.0 Connector	DigiKey (ID: 609-4613-1-ND)	1	0.41
	Red LED	Mouser (ID: 696-SMLFT0805SRCTR)	1	0.36
	Connector Receptacle	DigiKey (ID: WM3917CT-ND)	1	0.72
	Resistor 390 Ohm	DigiKey (ID: 311-390CRCT-ND)	1	0.10
	Toggle Switch	DigiKey (ID: CKN1783CT-ND)	1	7.48
			Total:	9.27
Dynamic PCB	Dynamic PCB Board	Fabricated PCB (File: Part8_Dynamic_PCB.zip)	1	0.20
	Arduino Nano 3.x	DigiKey (ID: 1050-1001-ND)	1	24.90
	Mini USB 2.0 Connector	DigiKey (ID: UE25BE5510H-ND)	1	0.83
	Right Angle Connector Header	DigiKey (ID: WM1743-ND)	1	0.25
	Resistor 100k Ohm 0805	DigiKey (ID: 311-100KCRCT-ND)	6	0.10
	Hall Effect Sensor	DigiKey (ID: AS5600-ASOMCT-ND)	1	4.51
	Capacitor 1 μF 0805	DigiKey (ID: 1276-1066-1-ND)	1	0.10
	Capacitor 10 μF 0805	DigiKey (ID: 445-7644-1-ND)	1	0.19
	Connector Receptacle	DigiKey (ID: WM4783CT-ND)	1	0.67
	Resistor 10k Ohm 0805	DigiKey (ID: RMCF0805FT10K0CT-ND)	2	0.10
	Motor Driver 20-QFN (3 × 3)	DigiKey (ID: 175-TMC6300-LA-TCT-ND)	1	3.84
	Resistor 120 mOhms 0805	DigiKey (ID: RL12S.12FCT-ND)	1	0.25
	Capacitor 0.1 μF 0805	DigiKey (ID: 478-KGM21NR71H104KTCT-ND)	3	0.10
			Total:	36.84

The total costs in Table 1 include raw materials and the fabrication of two bare PCBs (i.e., without any components). Purchasing fully assembled PCBs from the manufacturer will increase both costs and lead times. All prices are accurate as of February 2025.

**Table 2.** List of components necessary to build a motor-assisted commutator

Purpose	Item	Supplier/product number	Quantity	Price (US)
Motor-assisted commutator	Top Case	3-D Print (File: Part1_Top_Case.stl)	1	x
	M3 × 4 mm threaded insert	Adafruit (ID: 4255)	1	5.36
	PCB attachment	3-D Print (File: Part3_PCB_Attachment.stl)	1	x
	M2 × 4 mm threaded insert	McMaster-Carr (ID: 94180A312)	1	22.41
	Rotary Electrical Slip Ring	Taidacent (ID: 734831002031)	1	75.98
	Static PCB	Table 1	1	9.27
	M2 × 10 mm screw	Typically included with Rotary Electrical Slip Ring	4	x
	Dynamic PCB	Table 1	1	36.84
	Brushless DC Motor	DigiKey (ID: 1568-ROB-20441-ND)	1	29.95
	M1.6 × 4 mm screw	Maxx Model (ID: M1640HCS)	2	1.75
	Small Gear	3-D Print (File: Part11_Small_Gear.stl)	1	x
	Ball Bearing 6 mm shaft	McMaster-Carr (ID: 4668K122)	1	26.83
	Shield	3-D Print (File: Part13_Shield.stl)	1	x
	Cable Holder	3-D Print (File: Part14_Cable_Holder.stl)	1	x
	Magnet 5 mm × 1 mm	K&J Magnetics, Inc (ID: D301)	1	1.10
	M3-0.5 × 6 mm screw	McMaster-Carr (ID: 91290A111)	1	11.32
	Outer Gear	3-D Print (File: Part17_Outer_Gear.stl)	1	x
	M3-0.5 × 10 mm screw	McMaster-Carr (ID: 91290A115)	1	12.92
	M2-0.4 × 8 mm screw	McMaster-Carr (ID: 91290A015)	1	18.76
			Total:	254.24^a^

a A single commutator requires <5 of each screw. Without factoring in the cost of bulk screws, the cost of this commutator is approximately $210.

At the base of the motor-assisted commutator is the static PCB (Fig. 1*B*,*C*), which features two key ports: a Micro USB 2.0 and a 6-pin Molex PicoBlade connector. The Micro USB 2.0 port provides power to the device, while the 6-pin Molex PicoBlade connector enables reliable input–output communication for additional electronic devices.

At the opposite end is the dynamic PCB (Fig. 1*B*,*C*), which interfaces with the tether and adjusts in real time. This PCB integrates a built-in microcontroller (Arduino Nano 3.x, Arduino) that continuously monitors tether rotation using a magnetic field angle sensor (Hall sensor). This sensor detects shifts in the magnetic field to precisely estimate the angular position of the tether. Based on this data, the microcontroller activates a brushless motor to dynamically adjust the slip ring's orientation, ensuring smooth compensation for the mouse's movement (a detailed comparison of open-source commutators is provided in Table 3). The dynamic PCB also includes a 6-pin Molex PicoBlade connector, which is linked to its counterpart on the static PCB. This forms the input–output communication between both PCBs, allowing researchers to send instructions and receive real-time data during experiments while maintaining a stable, low-noise connection that is critical for sensitive behavioral and neural recordings.

**Table 3.** Comparative analysis of open-source active commutators

	Open-MAC	Open Ephys Commutator	FreiBox Commutator	MACHETE
Specifications				
Citations	Kapanaiah and Kätzel (2023)	Newman et al. (2024)	De La Crompe et al. (2023)	This paper
Open source	Yes	Yes	Yes	Yes
Motor mechanism	Stepper motor (NEMA14)	Stepper motor (NEMA11)	Stepper motor (NEMA17-03)	Brushless Motor (ROB-20441)
Driving mechanism	Stepper driver (TMC2208/9)	Stepper driver (TMC2130)	Stepper driver (A4988)	Motor driver (TMC6300)
Detection mechanism	Hall Effect Sensor (XF0342X20)	IMU/3D Tracking (HTC Vive)	Hall Effect Sensor (HAL830UT-A)	Hall Effect Sensor (AS5600-ASOM)
Onboard MCU	XIAO SAMD21 (ARM Cortex-M0+)	Teensy 4.0 (ARM Cortex-M7)	Arduino Uno Rev3 (A000073)	Arduino Nano 3.x (ATmega328)
Interface	Arduino-based	Arduino-based	Arduino-based	Arduino-based
Power supply	USB-C or DC (5–12V, 300 mA)	Micro-USB 2.0 (5V, 500 mA)	DC (12V, 600 mA)	Micro-USB 2.0 (5V, 500 mA)

### Assembly

**Figure 2.**
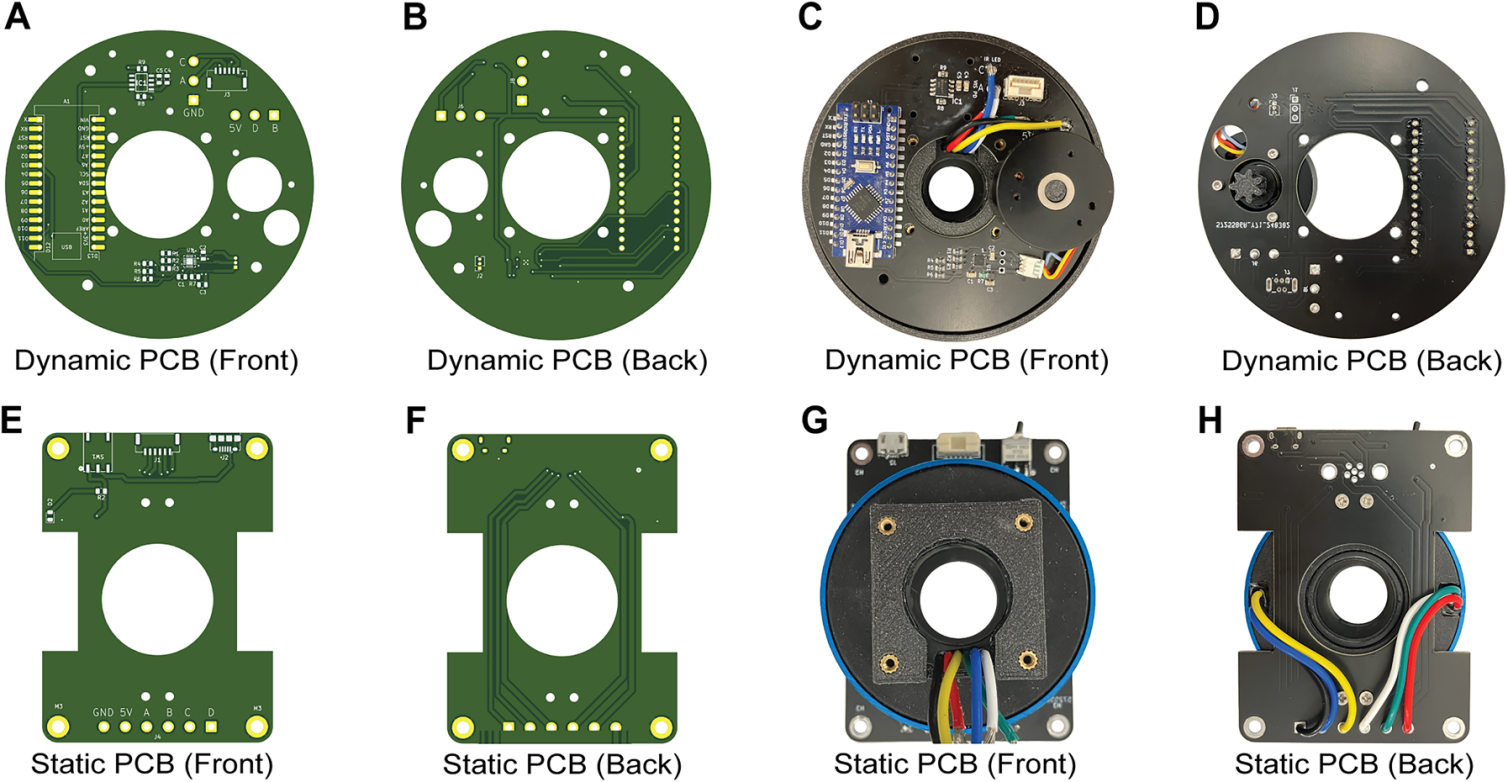
Overview of the static and dynamic PCBs. ***A***, ***B***, 3D model of the dynamic PCB, shown from the top (***A***) and bottom (***B***). ***C***, ***D***, Fully assembled dynamic PCB, includes the Arduino Nano 3.x, brushless motor, motor driver, Hall sensor, 6-pin Molex PicoBlade connector, and small gear. ***E***, ***F***, CAD model of the static PCB shown from the top (***E***) and bottom (***F***). ***G***, ***H***, Fully assembled static PCB, diagramming the slip ring with the PCB attachment, toggle switch, 6-pin Molex PicoBlade connector, and Micro USB 2.0.

Assembly of the MACHETE involves multiple steps that include the following: (1) soldering electrical components and connecting mechanical components onto the PCBs, (2) integrating the slip ring and PCBs with the 3D-printed parts, and (3) incorporating additional electromechanical components to fully construct and operate MACHETE. The digital design files for the 3D-printed parts, PCBs, and detailed assembly instructions are available on our GitHub repository: https://github.com/UNC-optics/MACHETE. The PCBs are assembled by soldering surface-mount components, including resistors, capacitors, and connectors. This can be completed manually with a soldering iron or streamlined using a reflow oven. Alternatively, preassembled PCBs can be outsourced using the PCB Gerber files. Illustrations of PCBs are shown in Fig. 2*A–H*.

The static and dynamic PCBs are soldered to the slip ring's six wires at through-holes for power (5V), ground (GND), and signal transmission (A, B, C, D). Each wire must connect to its identically labeled position on both PCBs to ensure continuous data and power transfer during rotation.

**Figure 3.**
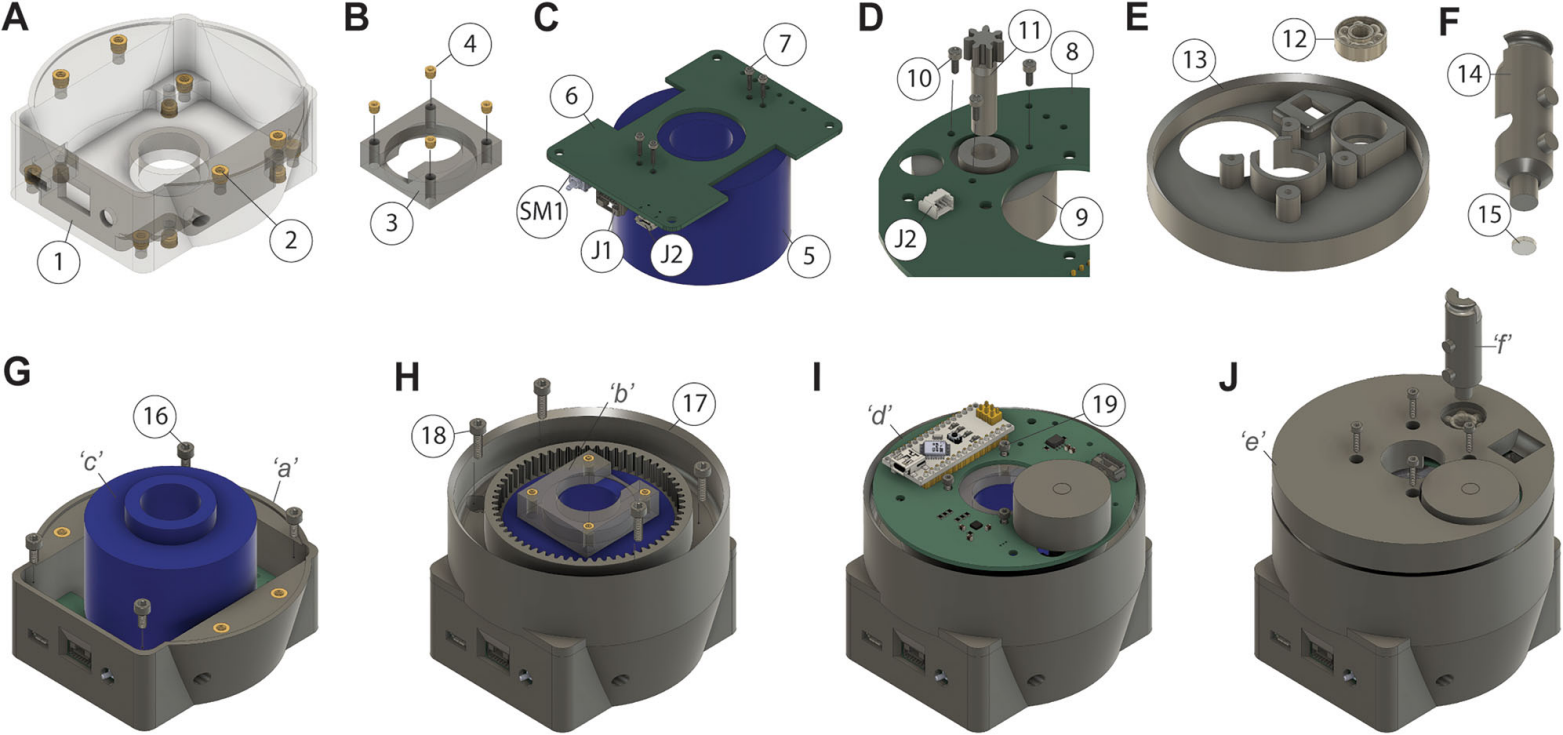
Step-by-step assembly of MACHETE. ***A***, The bottom base (part 1) of the commutator with the M3 × 4 mm threaded inserts (part 2). ***B***, The PCB attachment (part 3) with M2 × 4 mm threaded inserts (part 4). ***C***, Bottom of static PCB (part 6) attached to the slip ring (part 5) with M2 × 6 mm screws (part 7), showing the power toggle switch (PCB SM1), 6-pin Molex PicoBlade connector (PCB J1), and Micro USB 2.0 (PCB J2). ***D***, The small gear (part 11) inserted into the brushless motor (part 9) and glued into the place. The brushless motor (part 9) is secured to the dynamic PCB (part 8) using M2 × 4 mm screws (part 10), showing the right-angle connector (PCB J2). ***E***, A bearing (part 12) glued into the top case (part 13) of the commutator. ***F***, A magnet (part 15) glued to the bottom of the cable holder (part 14). ***G***, Static PCB assembly from ***C*** attached to the bottom base from ***A*** with M3 × 6 mm screws (part 16). ***H***, Middle case (part 17) attached to the bottom assembly using the threaded inserts in using M3 × 8 mm screws (part 18). ***I***, Dynamic PCB assembly from ***D*** placed on the nearly assembled commutator. ***J***, Top case assembly from ***E*** is secured to the PCB attachment with M2 × 8 mm screws (part 19) and the cable holder from ***F*** friction fit into the ball bearing.

The static PCB is screwed to the 3D-printed bottom base, while the dynamic PCB is screwed to a 3D-printed PCB attachment adapter that is glued to the top of the slip ring. An illustration of the gear-track system built into the 3D-printed middle case and cable-management system built into the 3D-printed top case are shown (Fig. 3*A–J*), and step-by-step assembly instructions can be found in our GitHub.

**Figure 4.**
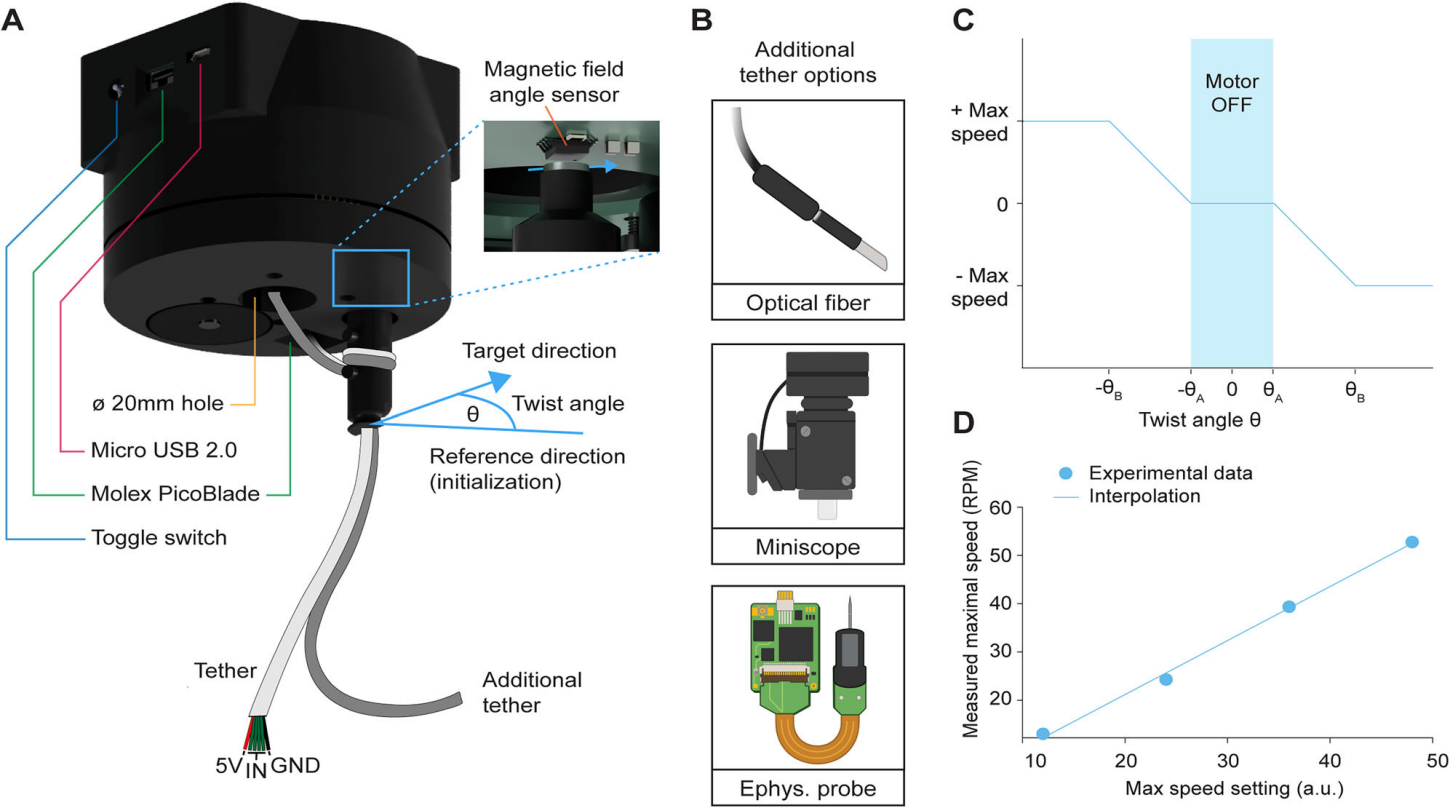
Magnetic sensing mechanism for active commutation. ***A***, Diagram of the magnetic field angle sensor, which detects cable twist by measuring the angle relative to an initial reference set at power-on (0°). When the twist angle, θ, surpasses a defined threshold, the commutator engages to counteract cable torsion. Highlighted are the different electromechanical components of the commutator, including the three wire ports, the magnetic field angle sensor, and the rotating cable holder. This system can accommodate a 6-wire tether which can be secured to the cable holder, as well as additional power and data cables. ***B***, The through-hole design allows for the use of external optogenetics, imaging, and electrophysiological devices during freely moving experiments. ***C***, The commutator's rotation speed increases proportionally with the twist angle, maintaining smooth cable alignment. ***D***, The system's performance is supported by a linear relationship between input speed and the maximum achievable RPM, demonstrating the high-speed correction capability of the gear mechanism and motor.

Once assembled, the commutator monitors and counteracts rotational changes in the tether. The brushless motor adjusts the dynamic PCB's orientation based on real-time data from a magnetic field sensor (Fig. 4*A*). The sensor detects the rotational movement of the tether via a magnet attached to the cable holder. This data is processed by a microcontroller on the dynamic PCB, which activates the motor to maintain precise cable alignment during neural modulation or recording experiments (Fig. 4*B*).

### Operation

To operate MACHETE, the microcontroller on the dynamic PCB is connected to a computer via a USB Type-B port, and the control code (available on our GitHub) is compiled and uploaded using Arduino IDE (version 2.3.2). Upon powering the system, the script initializes the brushless motor and the magnetic field angle sensor to establish a reference point for calibration (Fig. 4*A*). The motor operates with feedback-based velocity control, adhering to predefined voltage and frequency limits for optimal performance (Fig. 4*C*,*D*).

The system continuously monitors the magnetic field angle associated with the cable holder, tracking the rotational angle relative to the initial reference point. When the rotational angle exceeds a maximum and minimum threshold, the motor dynamically adjusts its speed proportional to the change in rotational angle to realign the tether, with a maximum velocity cap to prevent overcorrection (Fig. 4*A*,*C*). This real-time adjustment, coupled with continuous recalibration, effectively minimizes torque and mechanical strain by dynamically realigning the tether's alignment as needed (see Movie 1 for a direct comparison between a tethered mouse's movement with a motor-assisted commutator and a passive commutator).

### Animals

Adult, male and female mice C57BL/6J were group-housed under a reverse 12 h light/dark cycle (Zeitgeber Time 0 at 8:00 A.M.), with *ad libitum* access to food and water. Experiments were conducted in the dark cycle. All procedures were performed in accordance with the University of North Carolina at Chapel Hill's Animal Care and Use Committee's regulations.

#### Surgical attachment of baseplate

Mice were anesthetized with isoflurane (3–4% for induction and 1–2% for maintenance; Dechra Veterinary Products) and secured in a stereotaxic frame (David Kopf Instruments). A custom baseplate (9 × 6.5 mm) was secured on the skull using dental cement (C&B Metabond Parkell) to serve as a dummy head mount for a tethered connection to MACHETE. To test if mice tethered to our active commutator showed differences in behavior, we split mice into three groups (untethered vs 1× tethered vs 2× tethered, *n *= ∼5–8 mice/per group depending on behavioral test) and tested across several behavioral assays. A data cable, composed of five 30 AWG thick wires braided into a single cord, was used to tether mice to the commutator. In addition to this, the data cable was wrapped around a bifurcated 105-µm-diameter fiber optic cable connected to two implanted fibers in the skull and attached to the commutator.

### Behavioral testing

Prior to testing, all mice were habituated to researcher handling, and tethered mice were habituated to the tether in their home cage, during 10 min sessions across 3 consecutive days. Behavioral testing included the open field test, splash test, and three-chamber social test. During the behavioral assays, the untethered, 1× tethered, and 2× tethered mice were handled identically (i.e., experimenter also scruffed the untethered mice) when moving between their home cage and different behavioral arenas. The motor-assisted commutator was secured with four screws to an overhead optical breadboard, 58.42 cm from each of the testing arenas. A digital camera (DMK-22BUC03, The Imaging Source) with an infrared lens (XC0922LENS, Computar) was positioned above the testing arenas, and ANY-maze video tracking software (version 7.4, Stoelting) was used to collect video recordings and track mouse behavior for each experiment. To account for the effect of time, untethered and 1× tethered mice were alternated within each experimental session. Experiments with the 2× tethered mice for the open field test were performed on a different date with the same cohort of mice, whereas experiments for the splash test were performed on a separate cohort of mice.

#### Open field test

Mice were placed into an open-field arena (48 × 48 × 25 cm) and allowed to explore freely for a 10 min testing period. Total distance traveled (m) and time spent (s) in the center zone of the arena were quantified using ANY-maze software. The center zone was manually defined based on definitions commonly reported in the literature (Hall and Ballachey, 1932).

#### Splash test

The splash test was conducted using a 6% sucrose solution sprayed onto the dorsal coat of the mouse in its home cage (Yalcin et al., 2008). Following application, ANY-maze software was used to measure total distance traveled (m) within the home cage and to record videos of mouse behavior during a 5 min testing period. Grooming behavior was later analyzed through *post hoc* scoring of time spent performing grooming behavior (s) by a researcher using Behavioral Observation Research Interactive Software (BORIS; Friard and Gamba, 2016).

#### Three-chamber social test

The three-chamber social test was performed as described (Moy et al., 2004). Mice were initially placed in the center chamber of a three-chamber arena (61 × 30.5 × 30.5 cm) and allowed to explore freely for a 10 min habituation period. Following habituation, an unfamiliar juvenile conspecific (4–6 weeks old) was placed in a small cage in one corner of an outer chamber, while an identical empty cage was placed in the opposite outer chamber. During a subsequent 10 min testing period, ANY-maze software was used to measure total distance traveled (m) and time spent (s) in the social and nonsocial zones, defined by the presence or absence of the juvenile conspecific. The time spent in each zone was used to compute a social preference index using the formula: (Time in social zone − Time in non-social zone) / (Time in social zone + Time in non-social zone) (Moy et al., 2004).

### Statistics

Statistical analyses were performed using GraphPad Prism (version 10.2.2). Normality and variance equality were assessed using Shapiro–Wilk and the *F* or Brown–Forsythe tests, respectively. Parametric comparisons were conducted using *t* tests and one-way analysis of variance (ANOVA) with Bonferroni’s *post hoc* tests. Nonparametric analyses (Mann–Whitney *U*, Brown–Forsythe ANOVA followed by Dunnett's T3 for multiple comparisons, or Kruskal–Wallis followed by Dunn's tests) were used when assumptions were not met and when appropriate. Sample sizes were ∼5–8 mice per group. Data are shown as mean ± SEM. Statistically significant differences are marked as ****p < 0.0001 and nonsignificant differences are marked as (n.s.), *p* > 0.05.

### Data and code accessibility

The code/software generated in this paper is freely available online at https://github.com/UNC-optics/MACHETE. The code is available as Extended Data. Data generated and other information not available on our GitHub repository is available upon request to the lead contact and corresponding author, Jose Rodriguez-Romaguera (jose_rodriguezromaguera@med.unc.edu).

10.1523/ENEURO.0583-24.2025.d1 Extended Data The motor assisted commutator's functionality is encoded in MACHETE_Commutation_Script.ino, which can be uploaded to the Arduino Nano 3.x. Download Extended Data, ZIP file.

## Results

Tethered connections from implanted neural devices and auxiliary systems during behavioral experiments are prone to tangling, which can impose mechanical strain on mice. If unmanaged, this can disrupt naturalistic behavior, damage valuable equipment, or lead to data loss in critical experiments. MACHETE incorporates an automated angle-tracking system with a low auditory noise brushless motor to mitigate these challenges.

To demonstrate that MACHETE does not impede locomotor activity or other behavioral measures, and that these capabilities consistently extend across diverse experimental contexts, untethered, 1× tethered, and 2× tethered mice underwent the open-field test, splash test, and three-chamber social test. These assays are commonly used to assess behavioral endpoints relevant to psychiatric disorders because they are sensitive to changes in behavioral states (Eltokhi et al., 2020). Thus, we also assessed whether MACHETE introduces confounding neuropsychiatric-related phenotypes in mice.

**Figure 5.**
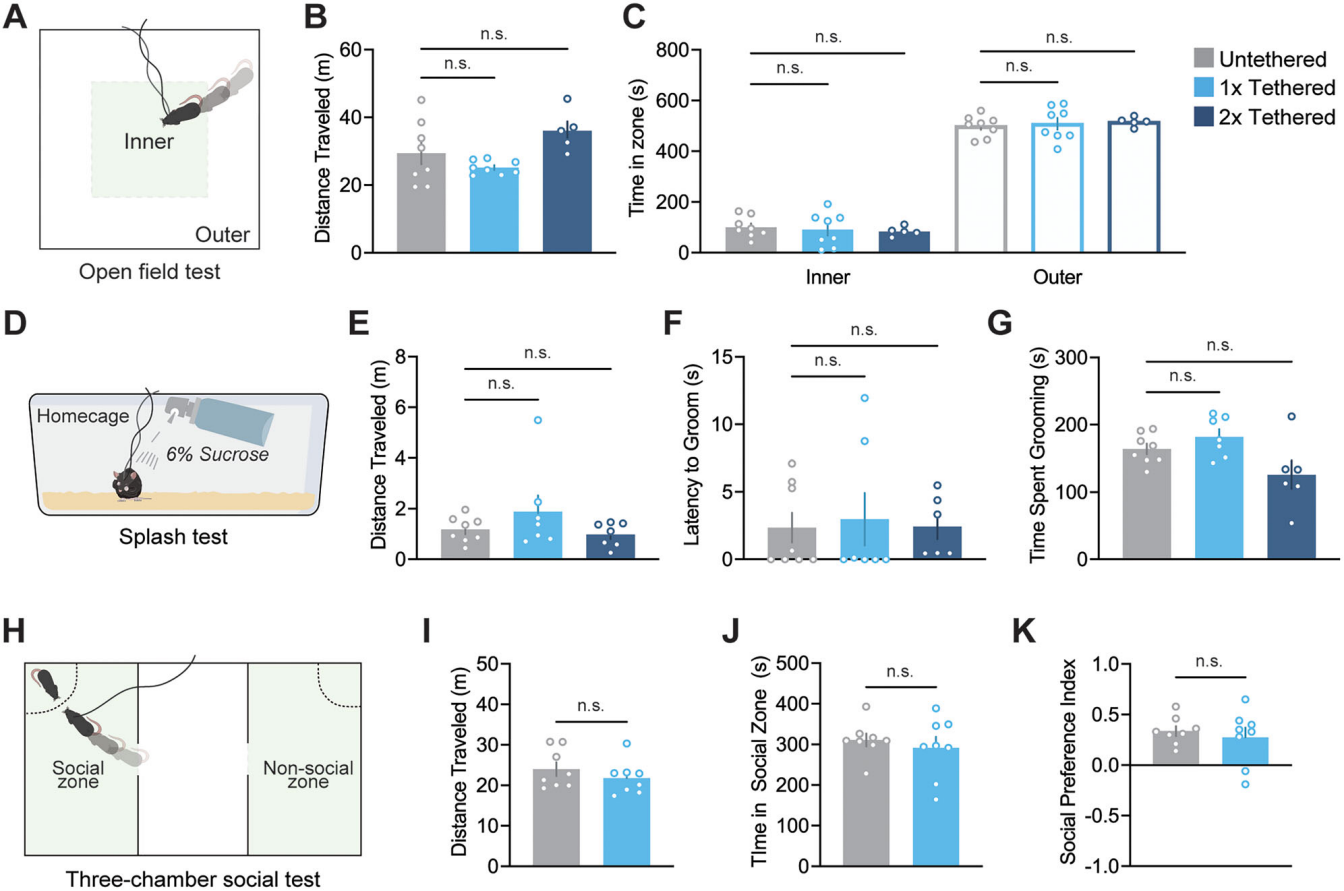
Behavioral testing with MACHETE shows retention of naturalistic behavior. ***A***, Schematic of arena used for the open field test with inner zone (green square) and outer zone labeled. ***B***, Comparison of total distance traveled in the open field test between untethered (*n* = 8), tethered (*n* = 8), and optical fiber (*n* = 5) mice. ***C***, Comparison of the total time spent in the inner and outer zones of the open field test between untethered (*n* = 8), tethered (*n* = 8), and optical fiber (*n* = 5) mouse groups. ***D***, Schematic depicting mouse being sprayed with 6% sucrose solution in their home cage during the splash test assay. ***E–G***, Comparison of total distance traveled, latency to initiate grooming behavior, and total time mice spent grooming between untethered (*n* = 8), 1× tethered (*n* = 7), and 2× tethered (*n* = 7 for ***F***, *n* = 6 for ***G*** and ***H***) mouse groups during the splash test. During these trials, one mouse did not initiate grooming behavior and therefore ***G*** and ***H*** are missing a single data point. ***H***, Schematic of three-chamber arena used in the three-chamber social test. ***I–K***, Comparison of total distance traveled, time in social zone, and social preference index between untethered and tethered mice (*n* = 8/group). Data are shown as mean ± SEM. ****p < 0.0001, n.s. = *p* > 0.05.

We performed the OFT, a widely used assay to assess general locomotor activity and anxiety-like behavior (Seibenhener and Wooten, 2015), to determine if tethering mice to MACHETE induces behavioral differences in a large, novel environment (Fig. 5*A*). During the 10 min testing period, we quantified total distance traveled and found no significant differences in total distance traveled between untethered and 1× tethered mice (adj. *p* = 0.71) and between untethered and 2× tethered mice (adj. *p* = 0.16; Fig. 5*B*). Since locomotor behavior was unaffected, we further analyzed time spent in the inner and outer zones of the OFT. We found that untethered, 1× tethered, and 2× tethered mice spent significantly more time in the outer zone compared with the inner zone (*F*_(5,36)_ = 148.8, *p* < 0.0001; Fig. 5*C*). Time spent in the outer zone, a measure of thigmotaxis (wall-hugging behavior), is used as an indicator of anxiety-like behavior (Seibenhener and Wooten, 2015). Therefore, we also compared if 1× tethered and 2× tethered mice had increased time in either zone relative to untethered mice. We found no significant differences between 1× tethered and 2× tethered mice in the outer zone (adj. *p* = 0.99, adj. *p* = 0.96) or inner zone (adj. *p* = 0.99, adj. *p* = 0.96; Fig. 5*C*).

**Movie 1.** Example movie providing a direct side-by-side comparison between mouse's movements in the open field arena with (left) and without (right) the active commutator. This movie demonstrates MACHETE's ability to detect rotational movement and correct for cable alignment during behavioral assays. The commutator detects changes in the mouse's direction based on changes in rotational angle and increases the rotational speed of the brushless motor proportionally to counteract twisting. On the left, this can be seen in the first minute of the experiment as the mouse explores the outer edge of the arena the commutator makes a full rotation in both directions to account for this initial exploratory behavior. Even as the mouse is stationary the commutator detects changes in direction, such as at 1 min and 25 s. In a 10 s period, the mouse pivots multiple times in the same spot and the commutator adjusts accordingly. The commutator's design also prevents twisting during more complicated behaviors, such as rearing. At 2 min and 22 s, a twist develops in the tether after the mouse stands on their hind legs against the wall to survey the arena three times in <10 s. A series of minor adjustments by the commutator untwists the tether and allows the mouse to continue exploring. On the right, when the commutator is powered off it functions as a passive commutator capable of movement through the internal ball bearing. However, as it moves the mouse can only generate enough torque to rotate the cable holder slightly. Just as the movie begins, a twist forms where the tether is connected to the baseplate. This twist is not corrected until 1 min and 30 s into the experiment, after a series of direction changes. This twist is a constant during the remainder of the experiment. Each twist in the tether reduces the total length of the tether, limiting the mouse's movement in the open field arena. Additionally, each twist of the wire can apply some tension, or pulling force, to the implanted baseplate which may cause discomfort and influence behavior. By the end of the movie, the original twist in the tether remains present.

Next, to evaluate if MACHETE impairs naturalistic behaviors such as locomotion and grooming in a familiar environment, we conducted the splash test in the home cage with both 1× tethered and 2× tethered mice (Fig. 5*D*). Untethered, 1× tethered, and 2× tethered mice were sprayed with sucrose solution on their dorsal fur, and then total distance traveled and duration of spontaneous grooming behaviors were quantified over a 5 min testing period. Our analysis revealed no significant differences in total distance traveled between untethered, 1× tethered, and 2× tethered mice (*p* = 0.37; Fig. 5*E*). Similarly, there were no significant differences between the latency to initiate grooming between untethered and 1× tethered mice (adj. *p* > 0.99) or untethered and 2× tethered mice (adj. *p* > 0.99; Fig. 5*F*). Furthermore, total time mice spent grooming showed no statistical difference between untethered and 1× tethered mice (*p* = 0.65) or untethered and 2× tethered mice (*p* = 0.12; Fig. 5*G*).

Lastly, we conducted the three-chamber social test with and without a tether to determine whether MACHETE interferes with behavior in a complex, novel environment (Fig. 5*H*). In terms of general locomotor activity, 1× tethered mice showed no significant differences in total distance traveled (m) compared with untethered mice (*t*_(14)_ = 0.99, *p* = 0.34; Fig. 5*I*). Next, we assessed whether 1× tethered mice exhibited differences in social behavior. We observed no differences between 1× tethered and untethered mice in time spent in the zone containing a social stimulus (s) (*t*_(14)_ = 0.62, *p* = 0.54; Fig. 5*J*). Furthermore, social preference scores did not differ between groups (*t*_(14)_ = 0.57, *p* = 0.58; Fig. 5*K*).

Together, these results demonstrate that MACHETE’s motorized commutation system effectively preserves natural movement patterns and behaviors across diverse contexts, supporting its capacity to maintain behavioral integrity in tethered experiments.

## Discussion

To gain a comprehensive understanding of brain function, it is essential to design experiments that incorporate ethologically relevant paradigms in freely behaving animals. While significant advancements have been made in the development of neural and electrical devices for use with mice in such experiments, the wired tethers required to operate these devices raise concerns about restricted movement due to tether entanglement and its impact on naturalistic behavior. These challenges are exacerbated when multiple devices, each requiring their own independent tether, are used within a single experiment (Das et al., 2023; Song et al., 2024). To address these issues, we developed a motor-assisted commutator specifically designed to manage wired tethers during freely moving behavior experiments.

Our device utilizes a brushless motor guided by a magnetic field angle sensor to dynamically reduce tension and prevent twisting of multiple tethers, which enabled us to record distinct animal behaviors, such as anxiety-like behavior, social interaction, and grooming activity, at levels comparable to untethered experiments. These behavioral assays are commonly used in neuroscience research to provide insights into anxiety, social, and stress-related behaviors under different conditions (Eltokhi et al., 2020). MACHETE also features a 20-mm-diameter through-hole design that is wide enough to allow electrophysiological devices, miniature microscopes, and optical fibers to pass through the center of the device unobstructed (Steinmetz et al., 2021; Zong et al., 2021, 2022). This structural feature enables seamless integration of optogenetic modulation tools with physiological recording setups, allowing for complex and varied experimental designs that include both neural stimulation and recording capabilities.

As advancements in electrophysiology and optical technologies support a higher number of recording channels and more intricate miniaturized optical components, there is an increasing demand for motor-assisted commutators to effectively manage the thicker coaxial and specialized wire cables required for enhanced data transfer (Sattler and Wehr, 2021; van Daal et al., 2021). For example, the coaxial tether used between the Miniscope (v4) and the Miniscope DAQ (v3.3) has an outer diameter of either 0.38 mm (equivalent to ∼27 AWG) or 1.14 mm (equivalent to ∼17 AWG). The Neuropixels (1.0/2.0) interface cable consists of two 26 AWG wires twisted together, resulting in an effective diameter close to 0.578 mm, which approximates a 23 AWG wire. The tether used in this paper is composed of five 30 AWG wires braided into a single cord, resulting in an effective diameter of ∼0.548 mm, also equivalent to 23 AWG. The effective thickness of the tether used in this study is comparable with that of the Neuropixels interface cable, while being slightly thicker than the Miniscope's smaller coaxial tether. The second tether, the optical fiber compatible with optogenetics experiments, has a diameter of 0.105 mm or ∼38 AWG. Behavioral experiments in the open field arena and home cage which used both tethers wrapped around one another had a larger effective thickness. MACHETE's active mechanism, namely, the brushless motor and motor driver, is potentially equipped to handle thicker coaxial and specialized wire cables, reducing the mechanical strain during freely moving experiments.

This motor-assisted commutator for freely moving behavior experiments is straightforward to implement. The commutator is constructed using readily available components and requires a basic understanding of soldering and 3D printing. This design aspect does result in some limitations. Since we are using 3D printer filament for major components of our device, such as the gear-track system and the cable holder, they are prone to wearing down with repeated use. Additionally, parts like the magnet and the ball bearing are glued into place. Eventually, this glue will disintegrate and will need to be reapplied. An additional limitation of this design is the height of our commutator. While shorter than existing commercial and open-source designs, the body of our commutator has a height of 70 mm and once the cable holder is fitted into place it extends to 99 mm. This can be a concern for experiments in smaller enclosures or behavioral arenas, where space is limited. Our design incorporates a slip ring for data transmission which has a height of 40 mm, which ultimately limits how compact we were able to design our device. With MACHETE, we aim to provide an accessible and adaptable tool to support neuroscience research aimed at understanding the relationship between neural circuits and behaviors. While our system was primarily tested with freely moving mice, it should be scalable to other small rodents or animals (e.g. zebra finches) that cannot generate sufficient torque to rotate most commutator systems (Fee and Leonardo, 2001; Liberti et al., 2017). Our motorized design can be integrated into a variety of experimental paradigms across model organisms, ensuring broad applicability across neuroscience studies. Open-source initiatives within the neuroscience community have significantly reduced financial and technical barriers, enabling a broader range of laboratories to perform complex behavioral assays that connect behavior with neural dynamics, thus fostering a more inclusive and innovative research environment.

## Synthesis

Reviewing Editor: Timothy Jarome, Virginia Polytechnic Institute and State University Decisions are customarily a result of the Reviewing Editor and the peer reviewers coming together and discussing their recommendations until a consensus is reached. When revisions are invited, a fact-based synthesis statement explaining their decision and outlining what is needed to prepare a revision will be listed below. The following reviewer(s) agreed to reveal their identity: R. Ryley Parrish, Kyle Parker.

Both reviewers agreed that the manuscript content is novel and well written. However, several concerns were raised that need addressed before the manuscript can be considered for publication.

1. Given the key takeaway from this manuscript is that MACHETE can be integrated into standard mouse behavioral neuroscience, the authors should provide an experiment demonstrating integration of multiple external devices e.g. optogenetic and electrical physiological recording integration.

2. Could the authors in some way provide a quantitative comparison of MACHETE in terms of auditory noise and ability to provide "tangle" free wired recordings compared to competing system? Does this system really provide a good solution for limiting tangles in wired applications?

3. Provide movies demonstrating MACHETE "in action" in one or more of the behavior tasks. This could provide a means to compare MACHETE to already available open-source systems. This will also demonstrate MACHETE detecting rotational movement and correcting for cable alignment.

4. For clarity, the reader would benefit from data describing the speed of the animals' movement in the behavioral tests. This can give a better depiction of how the animals move, especially in the open-field test in which animals may locomote differently depending on their location inside the arena, e.g "darting" behavior.

5. Authors should provide a schematic of the workflow for using their system. Pictures of this system being used on mice, ideally for electrophysiology, imaging, and optogenetic experiments would all be helpful in this workflow.

6. Please provide a clear estimate of the cost to build this device to include with table 1.

7. Authors should briefly discuss the scalability of their device

8. How well would this device work for other rodent models (short discussion would be helpful).

9. Authors should discuss the limitations of MACHETE. Where does it fall short and how can it be improved? Is it robust or prone to failure, etc.

10. Line 176-178: The authors should provide clear description regarding the handling of untethered mice prior to and during testing. Tethered mice are likely to be restrained for a short period of time to attach the commutator cable, yet the reader may assume all mice were treated similarly, including this restraint. The authors should edit this for clarity.

## Author Response

1. Given the key takeaway from this manuscript is that MACHETE can be integrated into standard mouse behavioral neuroscience, the authors should provide an experiment demonstrating integration of multiple external devices e.g. optogenetic and electrical physiological recording integration.

We thank the reviewers for this insightful comment. We agree that we should provide experimental demonstrations of the integration of external devices with our commutator. We ran two additional experiments, the splash test (ST) assay and open field test (OFT), with two Ø105 µm fiber optic cables connected to implanted fibers in the skull. We have revised "Figure 5. Behavioral testing with MACHETE shows retention of naturalistic behavior." to include the data from 7 mice in the ST assay and 5 mice in the OFT with both the original 5-wire tether and the two optical fibers connected to the commutator. The data from the ST shows no change in significance distance traveled for all 7 mice compared to the untethered mice. Latency to groom and time spent grooming showed no significant change in 6 mice, although one mouse failed to groom during the duration of the experiment. This failure to groom is outlined in the figure legend. For the OFT, distance traveled, and time spend in the inner and outer zone showed no significant change for the 5 mice tested. Overall, the addition of data with both the original 5-wire tether and two Ø105 µm fiber optic cables being managed by the commutator provides a more realistic validation of the device.

2. Could the authors in some way provide a quantitative comparison of MACHETE in terms of auditory noise and ability to provide "tangle" free wired recordings compared to competing system? Does this system really provide a good solution for limiting tangles in wired applications? The authors acknowledge the revision and agree with the reviewers that some quantitative comparison of MACHETE in terms of auditory noise and detangling of wires. In the original submission, on lines 77-78 we stated, "MACHETE employs a brushless motor to minimize noise and reduce torque, enabling smoother tether management during behavioral assessment.". After reassessing recent open-source commutator publications, we found no quantitative measure of auditory noise. Most claims of "low-auditory" noise from the literature cite the equipment used (i.e. stepper motor and silent stepper driver (Kapanaiah et al., 2023) being inherently noise-free compared to alternative electromechanical approaches. In our case, we used a brushless motor and silent stepper driver which, due to the absence of physical brushes which generate friction, are a more noise-free option. Small rodents especially, which exert lower loads and don't move at speeds which would lead to high-motor speeds, cause minimal vibrations.

In terms of the commutator's ability to provide a "tangle" free wired recording, we have provided a video recording in the extended data section which highlight the twisting and mechanical strain common in many behavioral tasks. Multimedia 2 is a side-by-side video that shows a mouse with a 5-wire tether attached to a 3D printed baseplate implanted to its skull becoming untangled as they move through the active mechanism of the commutator on the left-hand side. The second video on the right-hand side shows that when the sensors and motors are turned off, rendering it a passive commutator via the cable holder attached to a ball bearing, the tangling exerts force on the mouse and inhibits normal behavior. This video outlines the main function of the commutator, reducing twisting and tangling of the tether while retaining naturalistic behavior of untethered animals. We did not explicitly compare our commutator to other open-source or commercial commutators, but the analysis we ran with ANY-maze and BORIS provide insights on distance traveled, time in zones, time grooming, and sociability which are important metrics for qualifying the effectiveness of a commutator system.

3. Provide movies demonstrating MACHETE "in action" in one or more of the behavior tasks. This could provide a means to compare MACHETE to already available open-source systems. This will also demonstrate MACHETE detecting rotational movement and correcting for cable alignment.

We thank the reviewers for this suggestion. We have provided a file in the extended data section demonstrating the tethered mice during the open field test (OFT), with and without the assistance of the commutator. The mouse in the open field test is connected to the 5-wire tethered described in the manuscript. The video includes a view of the mouse moving in the open field and a view of the bottom of the commutator, allowing researchers to visualize the active mechanism. We have provided a direct comparison between the mouse's movements in the open field arena with and without the commutator. The recording of the mouse attached to MACHETE is mirrored vertically to provide a similar point of reference for the commutators relative to the arenas. While the mirroring changes the understanding of the direction of the mouse, it makes it easier to compare the active and passive mechanisms of the commutators as a response to direction change in the different experiments. These visualizations allow researchers to understand how the commutator detects and reacts to movements of the tethered mouse and how an absence of a commutator can change the mouse's behavior. These videos also allow researchers to directly compare the active mechanism of our system to other commutator systems, as mentioned by the reviewers.

4. For clarity, the reader would benefit from data describing the speed of the animals' movement in the behavioral tests. This can give a better depiction of how the animals move, especially in the open-field test in which animals may locomote differently depending on their location inside the arena, e.g "darting" behavior.

We thank the reviewer for this insight. An animals' movement during behavioral tests is defined not only by direction but speed. An animals' speed in the open-field is characterized by darting far more than in their home cage or in the three-chamber arena. In figure 5 we highlighted distance traveled as an important metric. This darting behavior is present in each arena, at variable rates. We are able to extract these speeds easily using AnyMaze. However, animal speed isn't necessarily driving commutator action as much as direction of movement. The Arduino code uploaded to MACHETE allows for automated detection of rotations based on the change in angle from the initial position. The motor changes its speed proportional to the change in rotational angle. Darting behaviors and other instances where the mouse's velocity increases don't necessarily result in a change of angle or twisting of the tether. For example, in the video we provide there are multiple instances where the mouse's speed increases as it enters and leaves the inner zone of the open field arena. During these times, the commutator doesn't detect a large enough rotational change, so there is no change in activity until the mouse changes direction.

Even at high speeds, mice and other small animals cannot generate enough torque to rotate the slip ring or other rotational device within a passive commutator to prevent twisting. Each of the open-source active commutators in table 3 (Kapanaiah et al., 2023; Newman et al., 2023; De La Crompe et al., 2023) use hall effect sensors to detect changes in rotational angle to initialize active commutation of tethers. Each of these commutators use a similar detection mechanism which prioritizes change in angle over speed.

5. Authors should provide a schematic of the workflow for using their system. Pictures of this system being used on mice, ideally for electrophysiology, imaging, and optogenetic experiments would all be helpful in this workflow.

We thank the reviewers for highlighting this gap in our manuscript. We have revised figure 4 to include a schematic of the workflow for using this system. This includes additional information on the placement of the micro USB 2.0 and Molex PicoBlade connectors, as well as a diagram of the additional power and data cables being fed through the 20 mm diameter through-hole which makes the commutator compatible with most commercial optogenetics, miniature microscopy, and electrophysiological devices. We highlight that these additional cables can be accommodated by the 3-D printed cable holder, alongside a 6-wire tether.

In Multimedia 2: MACHETE_Comparison_Video.mp4, we provide a visualization of the mouse using the original 5-wire tether. This depicts the tether attached to the custom 3D printed baseplate. For any configuration with additional cables, those cables would be similarly attached to the baseplate on one end and the cable holder on the other. The commutators design with the center through-hole allows additional cables to be wrapped around the cable holder and achieve the same level of commutation as the tether cable which is connected to the Molex PicoBlade connector at the bottom of the device.

6. Please provide a clear estimate of the cost to build this device to include with table 1.

We would like to thank the reviewers for this suggestion. We have revised both table 1 and table 2 to include a column for estimating the cost to build this device. Table 1 outlines the cost of building a single static and dynamic printed circuit board, while Table 2 factors in the costs outlined in Table 1 along with all other electromechanical components. Including these prices will help readers compare the cost of their existing equipment to building our device, as well as allowing them to factor in the total cost of building multiple devices. All prices were accurate as of February 2025.

7. Authors should briefly discuss the scalability of their device We thank the reviewers for this comment. In terms of scalability for different applications, the commutator can be applied to different freely-moving experiments which require data transmission and collection. For example, the leads of an electrocardiogram (ECG) or cables from other devices recording vitals and/or biometric data can be actively commutated. By either connecting the wires directly to our commutator to transmit the electrical signal to the data acquisition computer or fed into the through-hole to connect to the computer directly. In terms of scalability in terms of size, the open-sourced files located in the GitHub allow researchers to customize the commutator to their needs. The 3-D printed and PCB Gerber files can be scaled to larger sizes and edited to account for additional electronics or physical connections. Additionally, the open-source Arduino script allows for users to encode custom commands and integrate custom electronic devices both at the headplate and along the commutator. The limiting factor for scaling up this design is the size of the slip ring as we designed the commutator with a smaller slip ring to reduce the device's total size. We have added a statement about scalability in lines 353-358 in the manuscript.

8. How well would this device work for other rodent models (short discussion would be helpful).

We acknowledge the importance of this additional section and thank the reviewers for this comment. There are multiple instances of recent commutator papers that either test their device on other species such as zebra finches (Fee et al., 2001; Liberati III et al., 2017) or propose that using the system with other model organisms is possible (Roh et al., 2015; Barbera et al., 2020; Li et al., 2021). The brushless motor through the motor driver can achieve up to 2000 RPM. In figure 4, part C we highlight that the maximum speed setting of the commutator is 50 arbitrary units which correlates to over 50 RPM. The number of RPMs of the gear along the gear track to avoid tangling of the tether connected to a mouse is significantly smaller than the maximum speed achievable by the commutator. Given that our Arduino script allows us to precisely adjust speed according to the torsion of the cable holder, our device is compatible with any species which moves at speeds up to a little more than 50 RPMs. We have added a statement in lines 353-358 of the manuscript which outlines how this device can be used with other small rodents and other species.

9. Authors should discuss the limitations of MACHETE. Where does it fall short and how can it be improved? Is it robust or prone to failure, etc.

We thank the editor for outlining the need for a more descriptive limitations section. We have highlighted three potential limitations of our device. The first two are based on the use of materials that may degrade over time, namely 3D printer filament and super glue. The third final limitation is the height of the commutator, which can become a concern for experiments in smaller enclosures or behavioral arenas. We added a paragraph covering each of these limitations in the context of the literature in lines 340-350.

10. Line 176-178: The authors should provide clear description regarding the handling of untethered mice prior to and during testing. Tethered mice are likely to be restrained for a short period of time to attach the commutator cable, yet the reader may assume all mice were treated similarly, including this restraint. The authors should edit this for clarity.

We thank the reviewers for their insights. We acknowledge that not explicitly describing the handling of untethered mice prior to and during testing, it can be difficult for readers to understand the treatments of this cohort compared to tethered mice. During these behavioral experiments, the untethered mice were scuffed and handled in an identical manner as the tethered mice. Both the untethered and tethered mice had an implanted 3D baseplate and were handled when moving between their home cage and the behavioral arenas. We added a sentence covering the treatment of untethered mice in lines 179-181.
